# Purification of human butyrylcholinesterase from frozen Cohn fraction IV-4 by ion exchange and Hupresin affinity chromatography

**DOI:** 10.1371/journal.pone.0209795

**Published:** 2019-01-09

**Authors:** Lawrence M. Schopfer, Oksana Lockridge, Emilie David, Steven H. Hinrichs

**Affiliations:** 1 Eppley Institute, University of Nebraska Medical Center, Omaha, Nebraska, United States of America; 2 CHEMFORASE, Mont-Saint-Aignan, France; 3 Department of Pathology and Microbiology, University of Nebraska Medical Center, Omaha, Nebraska, United States of America; USDA-ARS, UNITED STATES

## Abstract

Human butyrylcholinesterase (HuBChE) is being developed as a therapeutic for protection from the toxicity of nerve agents. An enriched source of HuBChE is Cohn fraction IV-4 from pooled human plasma. For the past 40 years, purification of HuBChE has included affinity chromatography on procainamide-Sepharose. The present report supports a new affinity sorbent, Hupresin, for purification of HuBChE from Cohn fraction IV-4. Nine batches of 70–80 kg frozen Cohn fraction were extracted with water, filtered, and chromatographed on 30 L of Q-Ceramic ion exchange sorbent at pH 4.5. The 4% pure Q-eluent was pumped onto 4.2 L Hupresin, where contaminants were washed off with 0.3 M NaCl in 20 mM sodium phosphate pH 8.0, before 99% pure HuBChE was eluted with 0.1 M tetramethylammonium bromide. The average yield was 1.5 g of HuBChE from 80 kg Cohn paste. Recovery of HuBChE was reduced by 90% when the paste was stored at -20°C for 1 year, and reduced 100% when stored at 4°C for 24h. No reduction in HuBChE recovery occurred when paste was stored at -80°C for 3 months or 3 years. Hupresin and procainamide-Sepharose were equally effective at purifying HuBChE from Cohn fraction. HuBChE in Cohn fraction required 1000-fold purification to attain 99% purity, but 15,000-fold purification when the starting material was plasma. HuBChE (P06276) purified from Cohn fraction was a 340 kDa tetramer of 4 identical N-glycated subunits, stable for years in solution or as a lyophilized product.

## Introduction

Cohn fraction IV-4 is a by-product of commercial blood fractionation. This fraction is rich in human butyrylcholinesterase (HuBChE) [1–4]. The Human Gene Nomenclature committee assigned the name butyrylcholinesterase (gene BCHE) in 1990 to the enzyme that had previously been called pseudocholinesterase or plasma cholinesterase. Methods to purify HuBChE from Cohn paste on an industrial scale [3, 5, 6] were developed after the value of HuBChE as a bioscavenger of nerve agents was recognized [7, 8]. The Baxter Co. purified HuBChE from Cohn paste, using procedures developed at the Walter Reed Army Institute for Research. Safety of the pure HuBChE produced by Baxter under good manufacturing practices (GMP) conditions was tested in phase I clinical trials in humans. The clinicalTrials.gov identifiers are NCT00333515 and NCT00333528. Industrial-scale purification of HuBChE from Cohn paste is ongoing at Therapure BioPharma Inc. in Mississauga, Ontario, Canada.

An important reagent in the purification protocol is the procainamide affinity gel [9, 10]. Therapeutics intended for use in humans must be manufactured with GMP quality reagents. The Procainamide Sepharose 4 Fast Flow affinity gel is available as a GMP quality reagent from GE Healthcare. A new affinity sorbent, Hupresin, was introduced by Brazzolotto et al [11] for purification of recombinant HuBChE expressed by insect cells. Hupresin is currently not manufactured under GMP conditions. This is the first report of purification of HuBChE from frozen Cohn fraction IV-4 using affinity chromatography on Hupresin.

## Materials

Hupresin was purchased from CHEMFORASE, Mont-Saint-Aignan, France, emilie.david@chemforase.com. The ligand is a custom-synthesized hybrid of tacrine and huperzine [11, 12]. Hupresin is covalently bound to Sepharose 4B beads. Frozen Cohn fraction IV-4 paste, (Grifols Therapeutics, Clayton, NC 27520) was stored at -80°C. Paste dated 2014 was prepared with a different filter aid than paste dated 2015. Outdated volunteer human plasma was from the Nebraska Medicine hospital blood bank. Chemicals were from Fisher Scientific and Sigma-Aldrich.

The process for purification included the following equipment and supplies: Quikscale columns on wheels (Millipore cat# GS251511, 250 x 800 mm). Zeta Plus E16 dual zone capsule depth filter (3M Purifications Inc., Meriden, CT, cat# E16E07A90SP05A) and filter holder (model# 16EZA). Q-Ceramic HyperD F non-compressible ion exchange sorbent (PALL Corp. Port Washington, NY, cat# 20066–056). One thousand Liter stainless steel tank manufactured by Brand Metal Works, Bellevue, NE to our specifications. Pellicon 2 mini cassette, Biomax 30 kDa polyethersulfone (PES) screen type C, 0.1 M^2^, B30K C 0.1M2 (EMD Millipore Corp., Billerica, MA cat#SK1M012W01). Pellicon Mini Cassette Holder, 88cm^2^ @ 0.11 M^2^ stainless steel (EMD Millipore Corp., cat# XX42PMINI). Masterflex PharmaPure tubing, #L/S 18 CP, 5/16” inside diameter, 7/16” outside diameter (Cole-Parmer Instrument Co., Vernon Hills, IL SKU#06435–18). MasterFlex L/S pump (Cole Parmer, easy-load model# 77200–60). Pulse Dampener (Cole Parmer cat# 07596–20). Pressure gauge (Mid-States Supply Co. Inc., Omaha, NE, Ashcroft B-Series cat#35-1036-SD-15L-100). Overpressure cutoff switch (Mid-States Supply Co. Inc., Ashcroft B-Series cat# B4-28-B-XFS-60psi). Conductivity meter Dist 4 (Hanna Instruments, Woonsocket, RI, cat# HI98304). 0.45 μm centrifugal filter (Chrom Tech, Inc., Apple Valley, MN, cat# CTF-CA045-03). Sterivex-GP 0.22 μm sterile filter (Millipore Corp Cat No SVGPB1010). Polyacrylamide gels were poured in house. Laboratory Series Freeze Dryer (Millrock Technology, Kingston, NY, model# LD53S3). Lyophilization vials 4 x 2 ml 13 mm S/L FNT, (West Pharmaceuticals, Exton, PA).

### Ultrapure water

All aqueous solutions were prepared with ultrapure water that was purified through the following steps.

1. Potable tap water was passed through a water softener (Wood Brothers IND, Lincoln, NE cat# 2510-48K). This pre-processed water was pumped sequentially through steps 2 to 7.
2. Carbon filter (Wood Brothers IND cat#2510-.50C) removes salts and some organics.
3. PrePak filter (EMD Millipore cat# PRPK0L003) removes particulates.
4. ProGard activated-carbon filter with a 0.5 micron screen (EMD Millipore cat# PROGTLOSIUS) removes particulates and chlorine to protect the Reverse Osmosis membrane
5. ELIX System model 35 (EMD Millipore cat# ZLXS60035) includes
  - Reverse Osmosis membrane removes ions, dissolved organics, microorganisms, and particulates
  - Electro deionization unit captures anions and cations electronically
  - After the above steps, water goes to a 1000 L storage tank in the 5°C cold room. Water from the tank is passed through a Super Q polishing unit before use.
6. Super Q (EMD Millipore cat# ZFSQ11503) includes ion exchange filters, organic resin filters, and a UV light irradiation source. The UV-light system disinfects, reduces total oxidizable carbon, destroys ozone, destroys chlorine, and chloramine.
7. The final step is filtration through a 0.2 μm filter.
8. During periods of storage the water is cycled through the Super Q for 7 min every hour.

**Fig 1:**
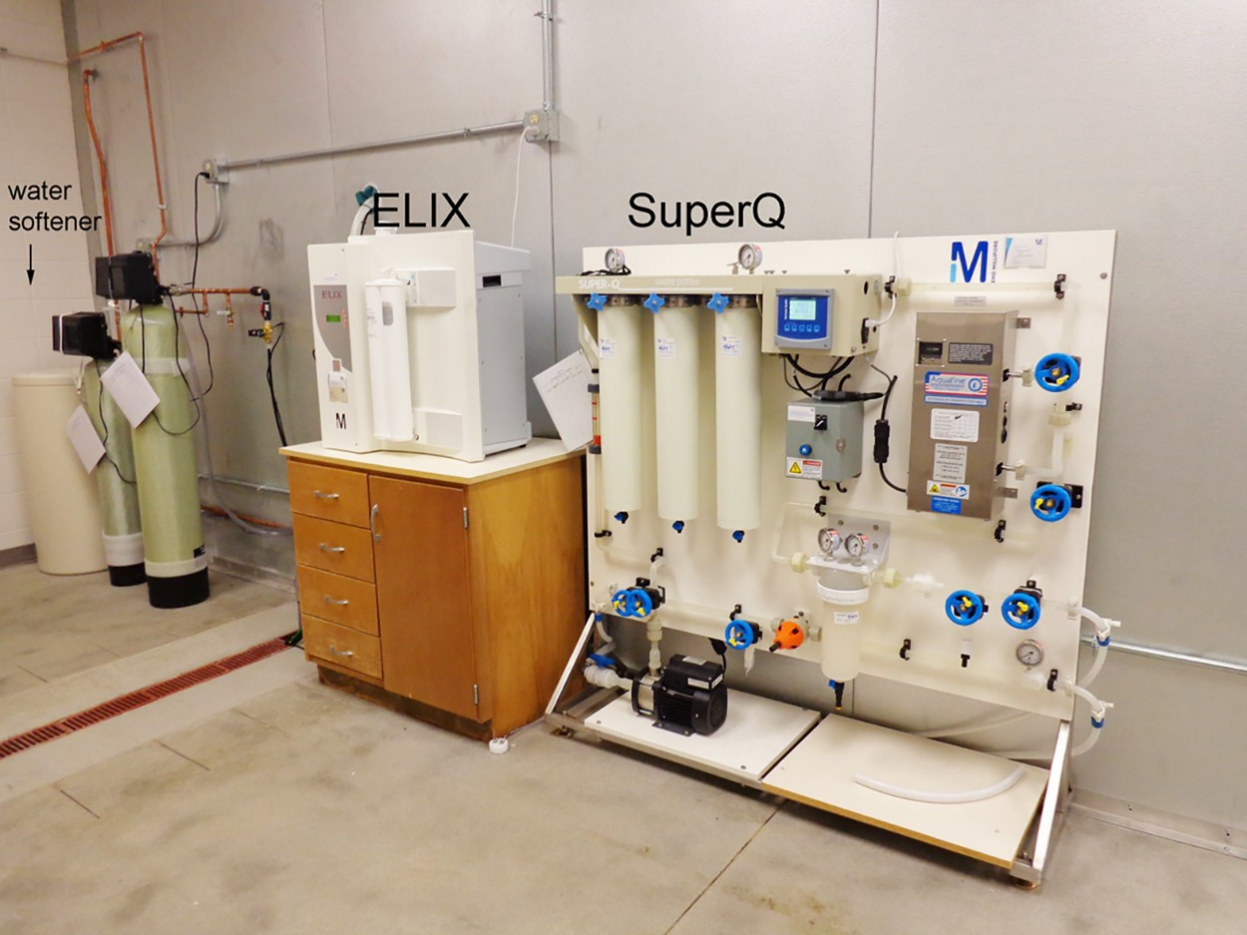
Components of the system that produces ultrapure water.

The ultrapure water had resistivity of 20 MΩ.cm at 5–10°C (equivalent to 18.2 MΩ.cm at 25°C), conductivity of 0.00 milliSiemens/cm (mS/cm), and was essentially free of endotoxin. A photograph of the ultrapure water purification system is shown in Fig 1.

### Priming the Zeta plus filter, the Q-Ceramic column, and the Hupresin affinity column

**Fig 2:**
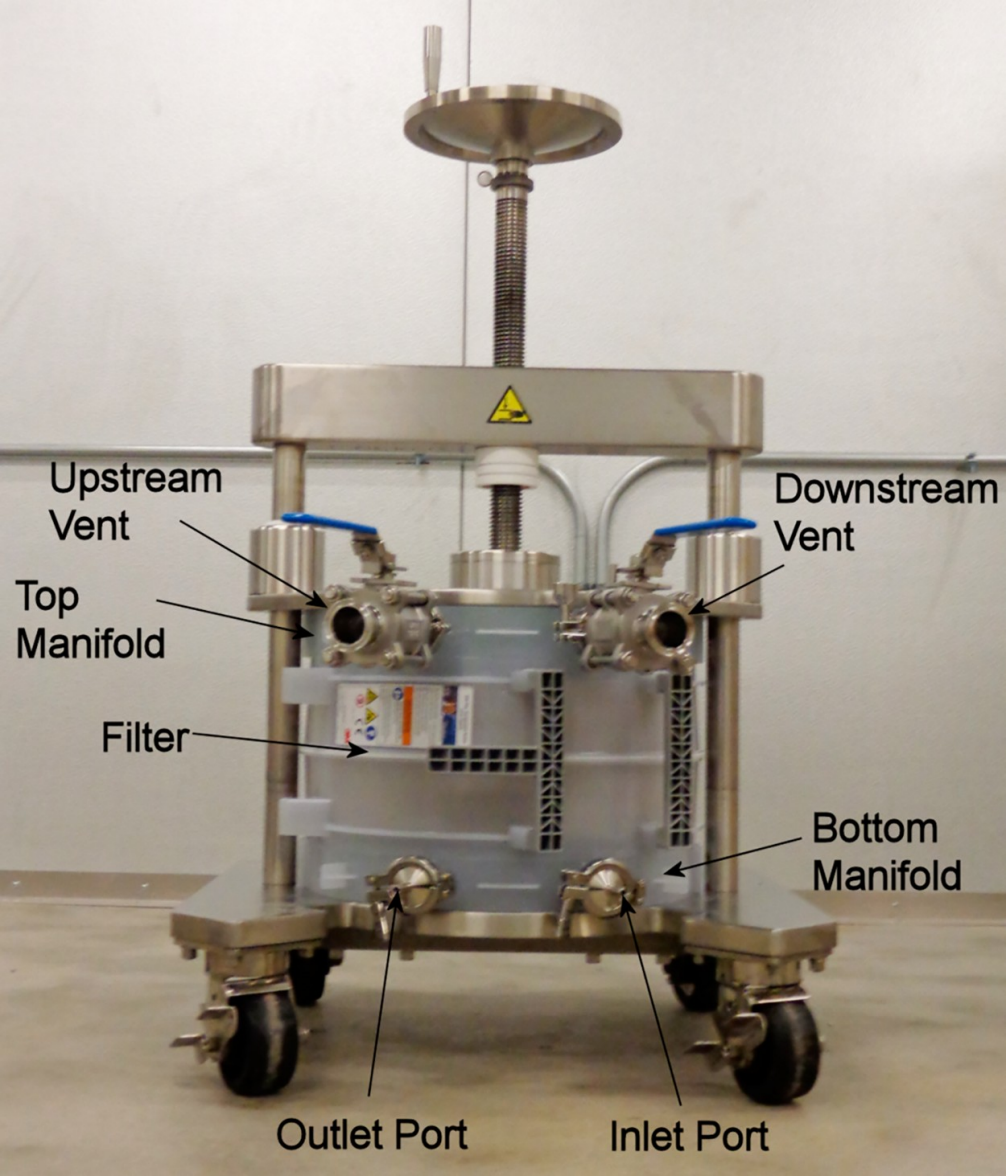
Zeta plus filter installed in its holder.

All procedures were performed in a cold room at 5°C. All solutions were prepared with ultrapure water. The Zeta Plus filter (Fig 2) was flushed with 120 L of 20 mM sodium acetate, 1 mM EDTA pH 4.5 to wash out yellow material, to remove air from the system, and to equilibrate the filter. Liquid was pumped through the filter at a pressure of 35 psi, well under the maximum recommended operating pressure of 50 psi for the filter.

**Fig 3:**
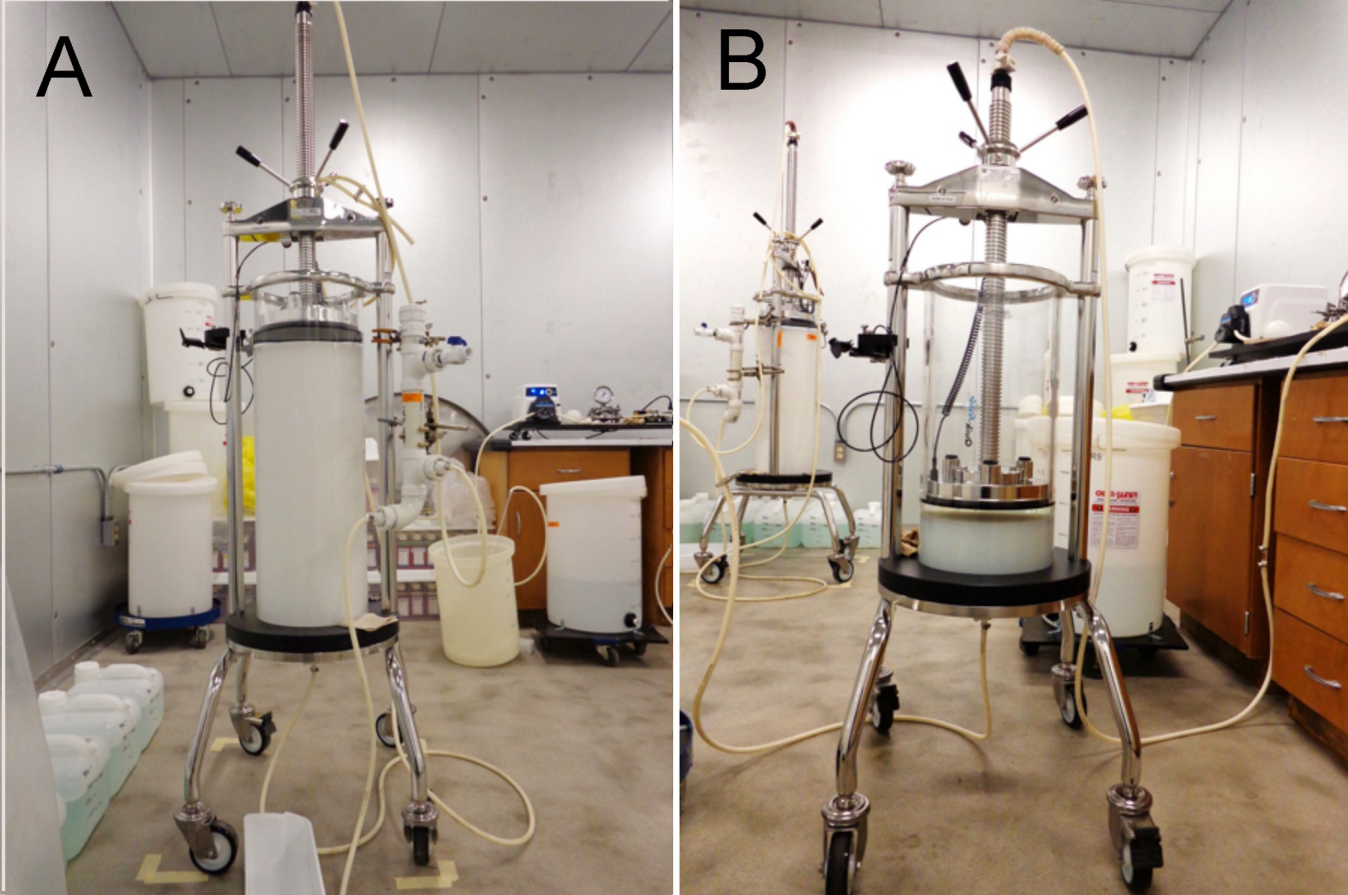
A) 30 L of Q-Ceramic ion exchange sorbent packed in a Quikscale column. B) 4.2 L of Hupresin packed in a Quikscale column; the Q-Ceramic column is in the background.

The Q-Ceramic ion exchange sorbent (30 L) packed in a Quikscale column (Fig 3A) was equilibrated with 200 L of 20 mM sodium acetate, 1 mM EDTA pH 4.5, conductivity 0.76 mS/cm, until the pH and conductivity of the effluent were the same as the starting buffer.

The Hupresin affinity gel (4.2 L) packed in a Quikscale column (Fig 3B) was equilibrated with 50 L of 20 mM sodium phosphate pH 8.0.

### Cohn fraction IV-4

All plasma donations used in the manufacture of Cohn fraction IV-4 paste were tested and found to be negative for Hepatitis B Surface Antigen, Anti-HIV-1/Anti-HIV-2, Anti-HCV and negative by FDA licensed HIV-1 and HCV nucleic acid testing. Plasma units were also screened for Parvo B19 by nucleic acid testing. Cohn fraction IV-4 from Grifols Therapeutics contains filter aid (purified diatomaceous earth) for separating microparticles from liquid. Frozen packets of Cohn paste were released by Grifols only after each lot was certified to be pathogen free. Frozen packets weighed 18 to 21 kg and were prepared either in Aug 2014 or in 2015. A change in the Grifols processing of fraction IV-4 occurred between 2014 and 2015. Cohn paste prepared by the 2014 process was a solid frozen block, whereas Cohn paste prepared by the 2015 process consisted of gravel-sized (1–3 cm) fragments. Yields of HuBChE from either process were the same. Packets were stored at -80°C for up to 3 years without affecting the yield of HuBChE.

### Cohn paste suspension, filtration, and loading on a 30 L Q-Ceramic column

**Fig 4:**
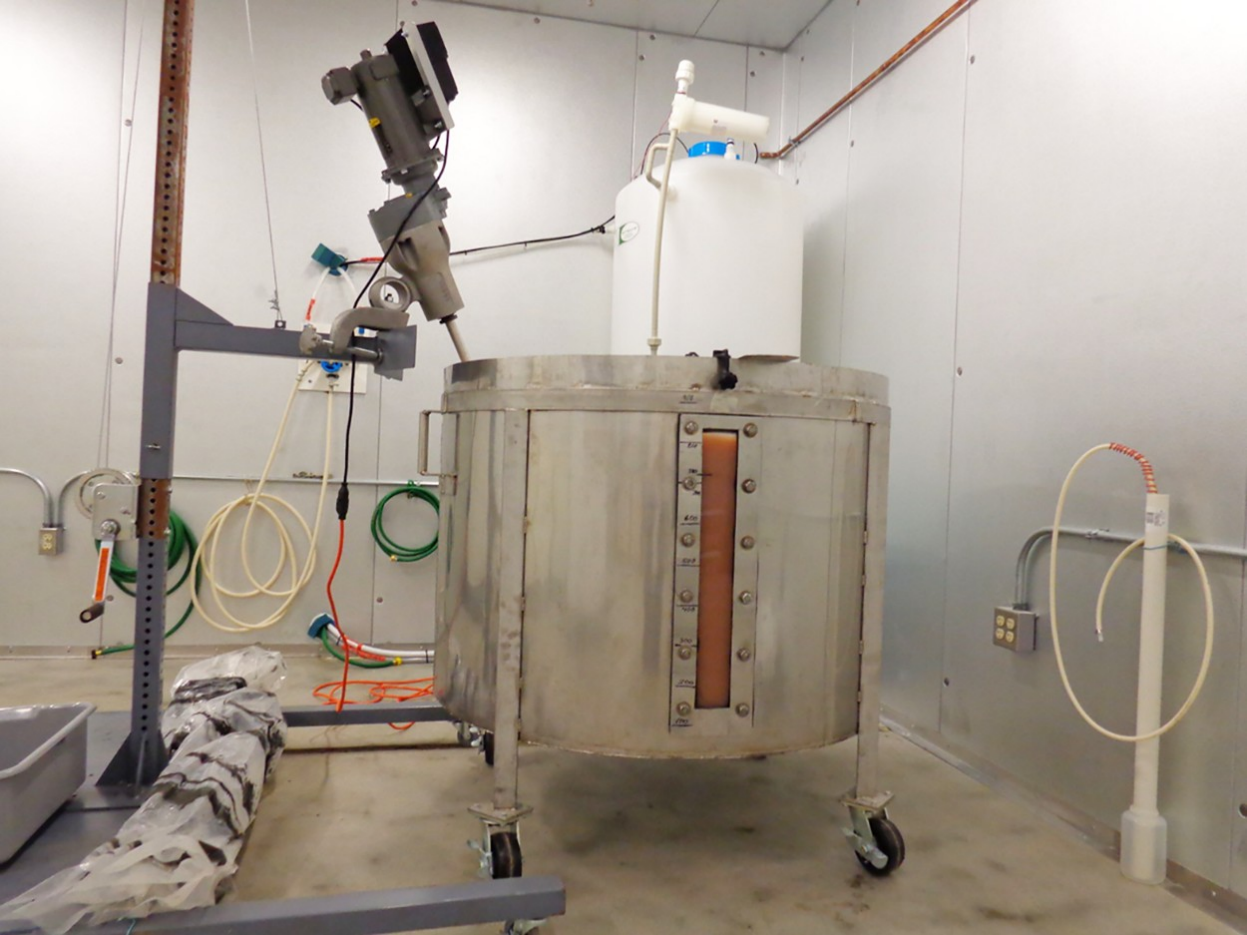
1000 L stainless steel tank containing 800 L of Cohn paste suspension. The brown color of the suspension is visible through the window on the side of the tank. The stirring paddle can be winch-lifted out of the tank. The white tank behind the stainless steel tank holds cold ultrapure water that is recycled for 7 minutes every hour.

Ultrapure water (720 L), to which had been added 304 g of solid tetrasodium EDTA, was cooled in a 1000 L stainless steel tank to 5°C. The 1 mM EDTA served as an antimicrobial agent. Frozen blocks of Cohn paste (80 kg) were added and the contents mixed with an overhead stirrer for 5 h to suspend the paste. The turbid suspension was acidified to pH 4.5 (pH measured at room temperature) with glacial acetic acid. Stirring was stopped and solids were allowed to settle to the bottom of the tank overnight. Fig 4 shows the 1000 L tank containing 800 L of brown slurry, being mixed with the overhead stirrer.

**Fig 5:**
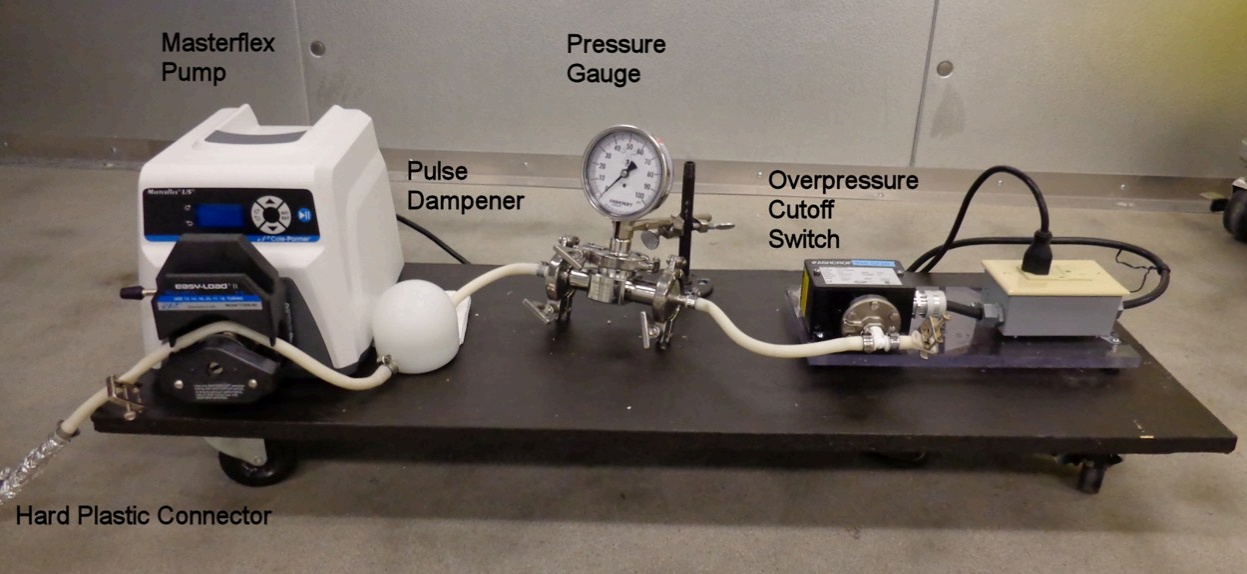
Pump assembly includes an Easy Load Masterflex pump, pulse dampener, pressure gauge, and overpressure cutoff switch.

The supernatant from the pH 4.5 extract was pumped at a flow rate of 590 mL per min through a Zeta Plus E16 dual zone capsule depth filter to remove fine particles that would otherwise clog the Q-Ceramic column. The effluent from the filter was pumped directly onto 30 L of Q-Ceramic HyperD F anion exchange sorbent packed in a Quikscale column. The pump was stopped after a total of 650 L of supernatant had passed through the filter and the Q-Ceramic column, leaving 150 L of sediment in the tank. The pump assembly is in Fig 5.

### Washing the 30 L Q-Ceramic column

The white Q-Ceramic ion exchange sorbent became brown when loaded with the filtered Cohn paste extract. The brown color was washed off with 750 L of 20 mM sodium acetate, 1 mM EDTA pH 4.5 at a flow rate of 550 mL/min. The first 100 L of wash buffer were pumped through both the Zeta plus filter and the Q-Ceramic column to recover BChE in the hold-up volume of the filter. The filter was taken out of line before the next 650 L of wash buffer were pumped through the Q-Ceramic column. At the end of the wash step the column had a faint blue color, and the effluent had an absorbance at 280 nm of 0.006.

### Elution of HuBChE from the 30 L Q-Ceramic column

The elution buffer, 50 mM sodium chloride in 20 mM sodium acetate, 1 mM EDTA pH 4.5, conductivity 5.77 mS/cm, was prepared in 120 L of ultrapure water. The buffer was pumped through the Q-Ceramic column at a flow rate of 550 ml per min. Fractions of 7.8 L were collected into plastic jugs. A sample from each fraction was tested for BChE activity. All the BChE activity eluted in 15 fractions. Fractions 1–6 were colorless, 7–8 were yellow, and 9–15 were blue to blue-green. The eluted BChE samples had pH 5.3±0.1. The majority of the activity appeared in fractions 8–10.

### Sanitation and cleaning the Q-Ceramic column

The Q-Ceramic column was washed and sanitized with 50 L of 0.5 M sodium hydroxide, pumped at a flow rate of 800 ml per min. The effluent was yellow, followed by blue-green, and yellow. After 1 hour the Q-Ceramic was immediately neutralized with 100 L of 200 mM sodium acetate, 1 mM EDTA pH 4.3 pumped at 800 ml per min. At the end of the neutralizing step, the effluent had the same pH 4.3 and conductivity 4.5 mS/cm as the starting buffer. At this point the pristine white color of the Q-Ceramic sorbent was restored. The column was equilibrated with 70 L of 20 mM sodium acetate, 1 mM EDTA pH 4.5, a buffer with a ten-fold lower sodium acetate concentration compared to the neutralization buffer, until the conductivity and pH of the effluent matched those of the equilibration buffer. The inlets and outlets of the column were clamped shut while the column was stored at 5°C.

### Loading Q-Ceramic eluate on 4.2 L Hupresin

Fractions 6–15 from the Q-Ceramic column had BChE activity. The 78 L of BChE in 50 mM sodium chloride, 20 mM sodium acetate, 1 mM EDTA pH 5.3 were pumped onto the 4.2 L Hupresin column directly without adjusting the pH, at a flow rate of 19 L per h. Higher flow rates were not used because they exceeded the pressure limit of 1.1 psi for Sepharose 4B beads. The overpressure cutoff switch in the pump assembly stopped the pump when the pressure exceeded 1.1 psi.

### Washing the 4.2 L Hupresin column

After the 78 L of BChE solution had loaded, the Hupresin column was washed with 40 L of 20 mM sodium phosphate pH 8, followed by 30 L of 0.3 M sodium chloride in pH 8 buffer. The wash buffer and 0.3 M sodium chloride effluents were discarded.

### Elution of BChE from 4.2 L Hupresin column

BChE was eluted with 12 L of 0.1 M tetramethylammonium bromide (TMA) dissolved in 20 mM sodium phosphate pH 8, followed by 10 L of 2 M sodium chloride in pH 8 buffer. Fractions of 1 L were collected into glass bottles. Fractions eluted off the Hupresin column were assayed for BChE activity and protein concentration. The activity was low in the first 4 fractions, highest in fractions 5–8, and decreasing in fractions 9 to 19.

### Cleaning the Hupresin column

The 4.2 L Hupresin column was washed with 2 M sodium chloride in 20 mM sodium phosphate pH 8 containing 0.01% sodium azide and stored in this buffer at 5°C. Hupresin was washed and sanitized with 0.1 M sodium hydroxide before run #3 and used an additional 7 times with no decrease in binding capacity. No additional washes with sodium hydroxide were performed.

### Concentration and buffer exchange in the Pellicon ultrafiltration/diafiltration device

The HuBChE in 0.1 M tetramethylammonium bromide and 2 M sodium chloride in 20 mM sodium phosphate pH 8 was reduced in volume from 19 L to 0.1 L by pumping the liquid through a Pellicon 2 Mini Cassette Polyethersulfone screen type C, 30,000 MW cutoff membrane at a flow rate of 1 L per 10 min. The 100 mL of clear BChE solution with a concentration of 20 mg/mL was collected into a glass bottle. The buffer in the BChE solution was changed to PBS by concentrating BChE to 50 mL in the Pellicon, diluting the BChE with 100 mL of freshly prepared PBS, and repeating the concentration and dilution step 15 times. The preparation became cloudy during this process.

### Filter sterilization

Cloudy material that developed during the buffer exchange step in the Pellicon was clarified by centrifugation followed by filtration through a 0.45 μ centrifugal filter. The particle-free HuBChE was filter-sterilized by pumping the protein solution through a Sterivex GP 0.22 μm filter unit into a sterile bottle. The sterile HuBChE in PBS was stored at 4°C.

### Endotoxin assay

Endotoxin levels in ultrapure water and HuBChE preparations were quantified with the kinetic chromogenic LAL assay [13]. Care must be taken when using this assay with HuBChE because the chromogenic substrate is hydrolyzed by native HuBChE to yield artificially high levels of endotoxin [13]. Accurate endotoxin levels in pure HuBChE were obtained by destroying the catalytic activity of HuBChE by heating the sample in a boiling water bath for 3 min. Boiled HuBChE samples were assayed for endotoxin. Boiling inactivates HuBChE without destroying endotoxin.

### BChE activity assay and protein concentration

Activity was measured at 25°C in 2 mL of 0.1 M potassium phosphate pH 7.0 in 1 cm path length quartz cuvettes containing 0.5 mM dithiobisnitrobenzoic acid and 1 mM butyrylthiocholine iodide, using a Gilford spectrophotometer interfaced to a MacLab computer. Increase in absorbance at 412 nm was recorded for 1 min and converted to μmoles per min using E_412nm_ = 13,600 M^-1^ cm^-1^. Units of activity are expressed as μmoles butyrylthiocholine hydrolyzed per min.

HuBChE that had been concentrated to 72,000 u/mL was assayed for activity after diluting the sample 10,000-fold as follows. Triplicate 5 μL aliquots were diluted into 495 μL of 1 mg/mL bovine albumin in phosphate buffered saline (PBS) to make triplicate 100-fold diluted samples. The 100-fold diluted HuBChE samples were further diluted by adding 5 μL to 495 μL of 1 mg/mL bovine albumin in PBS, for a final 10,000 fold dilution. The three 10,000-fold diluted HuBChE samples were assayed in triplicate using 10 μL of diluted HuBChE in a 2 mL reaction. Dilution into buffer containing albumin yielded 15% higher activity compared to dilution into buffer without albumin. Others have also found that addition of albumin to dilute solutions of HuBChE increases activity by minimizing sticking of HuBChE to the walls of the container [2, 6].

Protein concentration was measured from absorbance at 280 nm in a 4 mL quartz cuvette in a Gilford spectrophotometer. Absorbance at 280 nm of 1.8 represents 1 mg/mL of pure HuBChE.

### BChE tetramers and sialylation status visualized on nondenaturing polyacrylamide gels

Polyacrylamide 4–30% gradient slab gels, 0.75 mm thick, with a 4% stacking gel were poured in a Hoefer SE600 vertical slab gel apparatus. Electrophoresis at 4°C was at a constant voltage of 320 volts for 18 h. Gels to be stained for BChE activity were loaded with 0.002 to 0.005 units of BChE activity per lane; 0.002 units of BChE activity are present in 1 μL of the average human plasma. Gels were stained for BChE activity using the histochemical method of Karnovsky and Roots, adapted to polyacrylamide gels [14]. The substrate butyrylthiocholine iodide revealed brown-red bands for HuBChE. Partially desialylated HuBChE tetramers had reduced mobility due to loss of some of the negative charge on its glycans.

### Isoelectric focusing of HuBChE

Isoelectric focusing polyacrylamide gels were prepared and run according to the method of Garfin [15] in a Mini-PROTEAN Tetra Cell (BioRad 165–8003). The isoelectric focusing calibration broad pI kit (pH 3–10) and low pI kit (pH 2.5–6.5) were from GE Healthcare 17-0471-01 and 17-0472-01. The gel was stained with 0.04% Coomassie blue, 0.05% crocein scarlet 3B in 27% isopropanol, 10% acetic acid.

### SDS polyacrylamide gel

Polyacrylamide 4–30% gradient slab gels with a 4% stacking gel were poured in-house. Precast BioRad 4–20% gradient gels with no stacking gel were also used. Samples were denatured in a boiling water bath for 3 min in the presence of dithiothreitol and SDS. Gels were stained for protein with Coomassie blue.

### Liquid chromatography tandem mass spectrometry (LC-MS/MS)

The Hupresin-purified HuBChE was digested with peptide N-glycosidase F (PNGase F) to deglycosylate the protein before disulfide bonds were reduced with dithiothreitol and alkylated with iodoacetamide. The resulting material was digested with sequencing grade trypsin from Promega. Treatment with PNGase F not only removed the entire carbohydrate moiety from asparagine, but also converted Asn to Asp. This allowed positive identification of the location of N-linked glycans because the mass of Asp is 1 dalton higher than the mass of Asn. Deglycosylation had the additional benefit of allowing the Protein Pilot software to identify the amino acid sequences of the formerly glycosylated peptides. HuBChE in solution as well as Coomassie blue-stained gel bands were analyzed using this protocol.

A 5 μL aliquot of the digest representing approximately 5 μg of protein was analyzed on the 6600 Triple-TOF mass spectrometer using a data-directed fragmentation method, as described [16]. Data were searched against the UniProt protein sequence database (species Homo sapiens) using the Paragon algorithm v4.5 from Protein Pilot software v4.0 to match observed peptides to sequences in the database.

### Lyophilization

**Fig 6:**
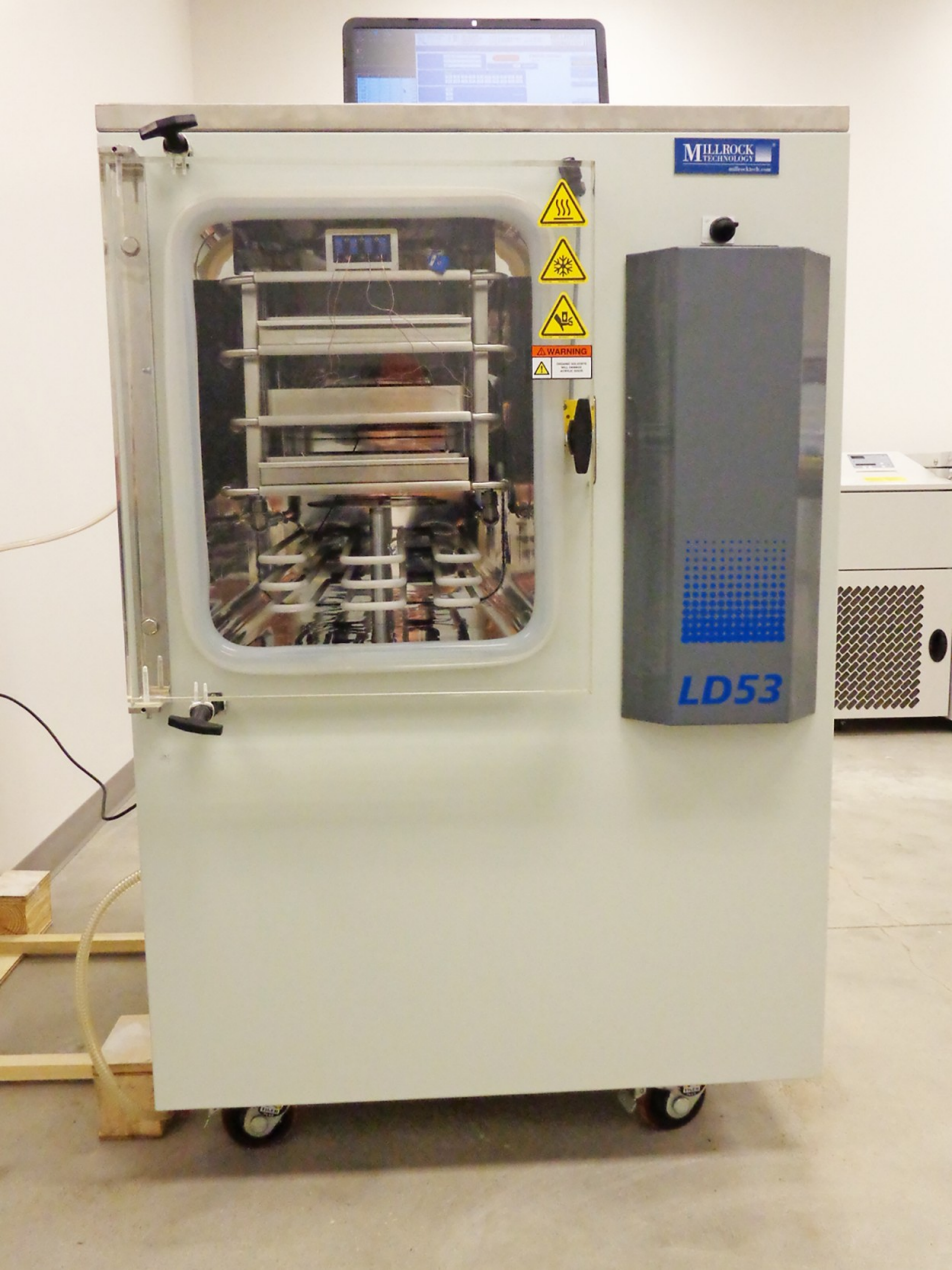
Shelf-type Freeze Dryer for lyophilizing HuBChE. Millrock Technology, model LD53S3, Kingston, NY.

HuBChE in 52 mM L-Histidine pH 6.0, 240 mM sucrose, 0.3 mM Tween-20 with an activity of 72,000 u/mL (which corresponds to 144 mg/mL; specific activity 500 u/mg) was aliquoted into glass lyophilization vials at 100 μL per vial. The samples were lyophilized in the shelf-type freeze dryer pictured in Fig 6. The vials of dry protein were capped with rubber stoppers, sealed with a metal caps, flushed with nitrogen gas and stored at 27°C. Periodically, samples were reconstituted with water and assayed for BChE activity.

## Results

### Extraction of HuBChE from Cohn paste

The Cohn paste was prepared by Grifols in 2014 and frozen. The decision to extract Cohn paste with 9 volumes of cold water (5°C) at pH 4.5 was based on comparison with 3 volumes of cold water. The 1:10 and 1:4 suspensions were adjusted to pH 4.0, 4.2, 4.5, 5.0, and 5.5 with glacial acetic acid. Turbidity and BChE activity were tested daily for 4–5 days. Solids settled out overnight at pH 4.5 from the 1:10 suspension, but required 48–72 hours at pH 4.2 and pH 4.0, and did not settle out at pH 5 and 5.5. Solids did not settle out at any pH from the 1:4 suspension. Settling out of solid material was cost-effective because it spared the Zeta filter (Fig 2) from clogging during filtration of paste extract.

**Fig 7:**
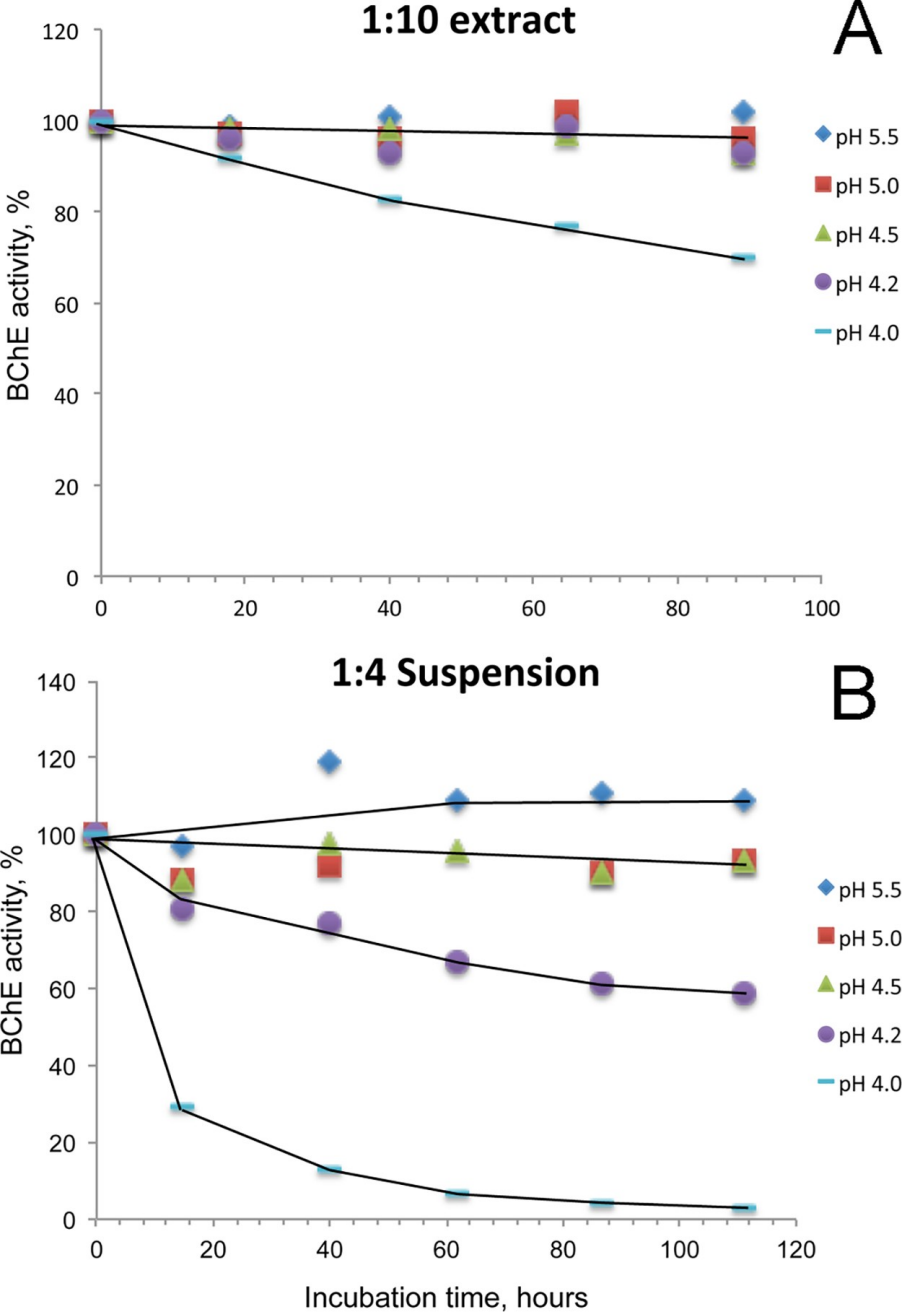
Stability of HuBChE activity in Cohn paste extracts as a function of pH and storage time. A) frozen paste extracted with 9 volumes of water, B) frozen paste extracted with 3 volumes of water. Extracts were stored at pH 5.5, 5.0, 4.5, 4.2, and 4.0 for 4 to 5 days at 5°C and tested daily for BChE activity.

The 1:10 extract retained BChE activity at pH 5.5, 5.0, 4.5, and 4.2, but lost 30% activity at pH 4.0 in 4 days (Fig 7A). The 1:4 suspension retained 93–110% BChE activity at pH 5.5, 5.0, and 4.5 but lost almost all activity at pH 4.0 after 5 days at 5°C (Fig 7B). It was concluded that filtration and HuBChE stability were optimal when Cohn paste was extracted with 9 volumes of water at pH 4.5 and 5°C.

Previous experience with ion exchange chromatography had shown that HuBChE binds to Q-Sepharose Fast Flow ion exchange sorbent in 20 mM sodium acetate, 1 mM EDTA pH 4.0–4.5 and elutes with 50 mM sodium chloride in the same buffer [10]. The buffer without salt had a conductivity of 0.5 mS/cm, buffer with 50 mM NaCl had a conductivity of 5.7 mS/cm, and the 1:10 extract had a conductivity of 1.5 mS/cm. This result suggested that the ionic strength in the 1:10 extract was low enough to allow HuBChE to bind to the Q-Ceramic sorbent at pH 4.5.

The protein concentration was 30–35 mg/mL in the 1:10 extract before filtration and 25–30 mg/mL after filtration, measured with the bicinchoninic acid protein assay.

### Yield and purification on Q-Ceramic ion exchange

Approximately 80 kg of frozen Cohn paste per batch was processed 9 different times as summarized in Table 1. The initial extract from the average 80 kg batch contained 4,300,000 units of BChE activity corresponding to 8.6 g of HuBChE. About 40% of the HuBChE activity bound to 30 L of Q-Ceramic at pH 4.5, while the remainder passed through the Q-Ceramic column without binding. This loss of HuBChE was not due to saturation of the Q-Ceramic sorbent. The HuBChE activity that passed through the Q-Ceramic could not be recovered. The unbound effluent was discarded. Bound HuBChE was eluted from the Q-Ceramic column with 50 mM NaCl in pH 4.5 buffer.

**Table 1:** Summary of HuBChE yield from Cohn paste extract purified on 30 L of Q-Ceramic^a^.

	Start Date	Cohn paste, kg	BChE ^b^ start, g	% BChE lost in pass-thru	BChE ^b^ eluted, g	BChE yield %	Cohn paste date ^c^	Paste age, mo^d^
1	14Apr2015	82.2	7.5	64	1.2	16	2014	8
2	26Jun2015	68.7	3.1	43	0.98	31	2015	3
3	10Aug2015	82.3	8.6	68	2.66	31	2014 & 2015	12
4	25Sep2015	80.0	8.9	66	2.17	24	2014	13
5	17Oct2016	80.0	8.60	60	2.62	31	2014	26
6	6Nov2016	80.1	7.88	59	2.65	34	2014	27
7	15Dec2016	80.0	8.10	59	2.52	32	2014	28
8	16Jan2017	82.1	9.53	59	2.25	24	2014	29
9	16June2017	80.6	9.48	62	2.59	27	2014	34

^a^ Capture on Q-Ceramic was performed at pH 4.3–4.5.

^b^ Yield in grams is calculated from the total units divided by 500,000 units/g.

^c^ Grifols used a different filter aid to prepare Cohn paste dated August 2014 than for Cohn paste dated March 2015.

^d^ Frozen Cohn paste was stored at -80°C.

Two steps in the purification protocol were accompanied by large losses of HuBChE activity. About 20% of the initial activity was lost in the settled pellet. About 60% of the remaining HuBChE passed through the Q-Ceramic column without binding. This calculates to 2.7 g bound out of an initial 8.6 g of starting HuBChE (8.5 g x 0.80 = 6.8 g applied to Q-Ceramic; 6.8 g x 0.40 = 2.7 g bound). Essentially all of the HuBChE that bound to Q-Ceramic (average range of 2.2 to 2.6 g) was recovered. The average overall yield from the nine preparations listed in Table 1 was about 30%. Further purification over Hupresin followed by concentration and buffer exchange caused minor additional losses.

The Grifols company prepared Cohn paste with a different filter aid in the year 2015 than in the year 2014. The yield of BChE was about 30% for both processes.

Runs 2–9 in Table 1 used paste that had been stored 3 to 34 months at -80°C. Yields were reproducible and did not diminish during the prolonged storage time.

This was a reassuring result because small scale studies had suggested that storage temperature was a critical factor in retention of HuBChE activity. We found that paste stored 1 day at 4°C lost all HuBChE activity, and paste stored at -20°C retained HuBChE for 7 weeks, but lost 90% of the HuBChE activity after 12 months.

### Binding capacity of Hupresin

We loaded 300,000 units of HuBChE activity per liter of Hupresin. This loading protocol minimized loss of HuBChE in the wash with 0.3 M sodium chloride. Hupresin easily bound higher quantities of HuBChE, but when higher quantities were loaded, a higher percentage of HuBChE was lost in the wash with 0.3 M sodium chloride.

### Yield and purification on 4.2 L Hupresin followed by ultrafiltration/diafiltration (UF/DF) and sterile-filtration

Hupresin purification yields are shown in Table 2 for 5 preparations. Earlier preparations are not listed because they preceded purchase of a second Quikscale column; repeated chromatography on smaller volumes of Hupresin was used for earlier batches. Table 2 shows that on average 1.8 g (85%) of the HuBChE activity loaded (2.1 g) on 4.2 L Hupresin was recovered in the eluate. Ultrafiltration/diafiltration (UF/DF) in the Pellicon followed by sterile filtration yielded an average of 1.5 g of pure HuBChE from the original 80 kg of frozen Cohn paste.

**Table 2:** Q-eluate ^a^ further purified on 4.2 L Hupresin, UF/DF in Pellicon, sterile-filtered.

Date	Q-eluate, BChE, g	BChE off Hupresin, g	Units/mg	BChE g after UF/DF & sterile filter
26oct2016	2.0	1.9	500	1.6
15nov2016	1.8	1.8	490	1.5
23dec2016	2.1	1.7	505	1.3
24jan2017	2.2	1.6	500	1.6
18june2017	2.6	1.9	500	1.5
Mean±S.D.	2.1±0.2	1.8±0.1	499±5	1.5±0.1

^a^ The term Q-eluate refers to protein eluted from Q-Ceramic sorbent with 50 mM sodium chloride in 20 mM sodium acetate, 1 mM EDTA pH 4.5.

The mean specific activity of pure HuBChE from 5 batches of frozen Cohn paste was 499±5 units/mg. We have chosen to use 500 units/mg as the specific activity of pure HuBChE purified from frozen Cohn paste. Our units of activity are defined in assays that use 1 mM butyrylthiocholine in 0.1 M potassium phosphate buffer pH 7.0 at 25°C. Activity assays by Sigma-Aldrich are performed at a higher temperature (37°C) and higher pH (pH 8.0). We found that 1 unit of BChE activity measured under our conditions corresponded to 4.2 units of BChE activity under Sigma conditions.

HuBChE extracted from Cohn paste was 0.1% pure (Table 3). Chromatography on Q-Ceramic pH 4.5 increased the average purity to 4% with a range of 0.3 to 12.4% pure depending on the fraction. Fractions that eluted late from the Q-Ceramic column had the most blue-green color and were the least pure. Chromatography on Hupresin increased HuBChE purity to 99%. There were no side-fractions from the Hupresin eluent because all fractions had the same specific activity of 500 u/mg.

**Table 3:** Purity of HuBChE after chromatography on Q-Ceramic and Hupresin.

	units/mg	% purity	Fold purification
Cohn extract	0.5	0.1	1
Q-Ceramic	20.3 (1.4–65)	4 (0.3–12.4)	40
Hupresin	500	99	1000

### Sanitation of Hupresin with sodium hydroxide

Hupresin retained its binding capacity for HuBChE after being briefly washed with 0.1 M sodium hydroxide and neutralized with 0.1 M phosphate pH 6.4. The sodium hydroxide wash was performed before run #3 (Table 1). Subsequent use of the same Hupresin successfully purified HuBChE from 7 additional 80 kg batches of Cohn paste.

A small scale trial tested the effect of sanitation with 0.1 M sodium hydroxide on the binding of BChE from 100 mL of human plasma by 2 mL Hupresin. We reported that 7 rounds of sanitation had no adverse effect [17].

### Other proteins in Cohn fraction IV-4

The following information was obtained by LC-MS/MS analysis of trypsin digested proteins extracted from SDS polyacrylamide gel slices taken from gradient gels used to separate the extract of Cohn fraction IV-4. The most abundant proteins in Cohn fraction IV-4 extract were transferrin P02787, albumin P02768, and BChE P06276. Substantial amounts of haptoglobin P00738 and hemopexin P02790 were also present. Albumin and transferrin passed through the Q-Ceramic column at pH 4.5 without binding. Haptoglobin was present in the Q-eluate, but passed through Hupresin during sample loading. Blue Q-eluate fractions contained ceruloplasmin P00450. Prekallikrein activator (PKA) was not detected in 99% pure HuBChE by mass spectrometry, though it was detected in the pass-thru material that failed to bind to Q-ceramic sorbent.

### Purity and size of purified HuBChE on SDS polyacrylamide gel

**Fig 8:**
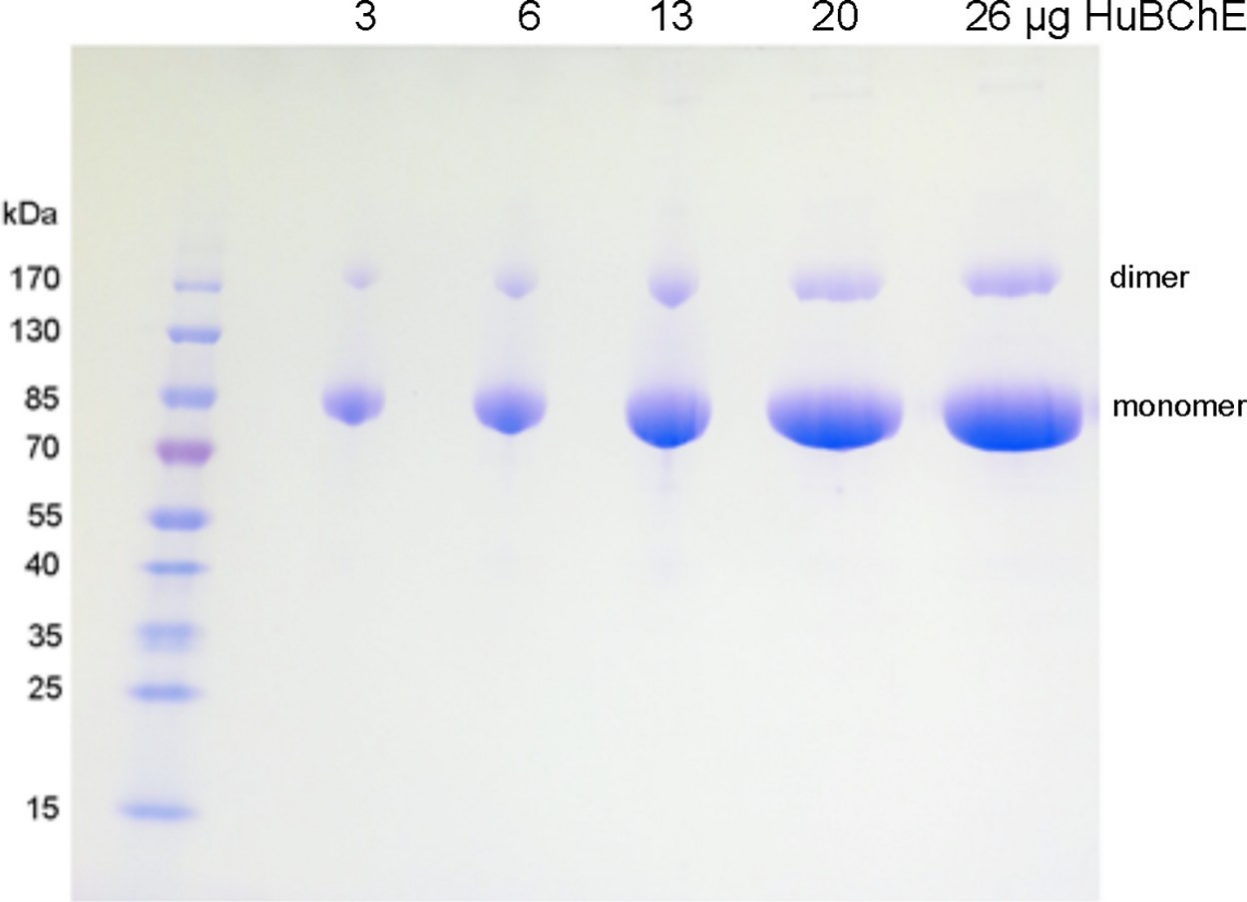
SDS polyacrylamide gel of 99% pure HuBChE purified by the method described in this report. HuBChE monomers are at 85 kDa and nonreducible dimers are at 170 kDa.

The molecular weight and purity of purified HuBChE concentrated to 25 mg/mL are shown in Fig 8. Samples were reduced with dithiothreitol in the presence of SDS in a boiling water bath before loading 3 to 26 μg of protein per lane on the SDS polyacrylamide gradient gel. The most intense band was the 85 kDa monomer of HuBChE. A nonreducible HuBChE dimer at 170 kDa was present as a minor band.

The Coomassie blue stained band at 170 kDa was cut out, digested with trypsin and analyzed by LC-MS/MS. Mass spectrometry confirmed that the band at 170 kDa is HuBChE. A more detailed examination of the MSMS fragmentation data from the mass spectrometer provided preliminary evidence for isopeptide bonds that could be responsible for the non-reducible dimer. Further work is currently underway to confirm these preliminary observations.

The molecular weight of 85 kDa per subunit is consistent with the known structure of HuBChE in plasma. The native enzyme is a tetramer of 4 identical subunits, each consisting of 574 amino acids and 9 N-linked glycans [18]. Two subunits are joined through a disulfide bond at Cys571 to form a dimer [19]. Each tetramer is a dimer of dimers. In addition, each tetramer contains a noncovalently-bound polyproline-rich peptide 2 to 5 kDa in size [20, 21]. The polyproline-rich peptide interacts with the C-terminal tetramerization domain of BChE to assemble 4 subunits into a stable tetramer. The polyproline-rich peptide is detectable by mass spectrometry in extracts of denatured tetramers. The cryo-EM structure of the HuBChE tetramer confirms the presence of a polyproline-rich peptide in the center of a super helix in the C-terminal tetramerization domain [22, 23].

### Sialylated, tetrameric HuBChE on nondenaturing gels stained for BChE activity

**Fig 9:**
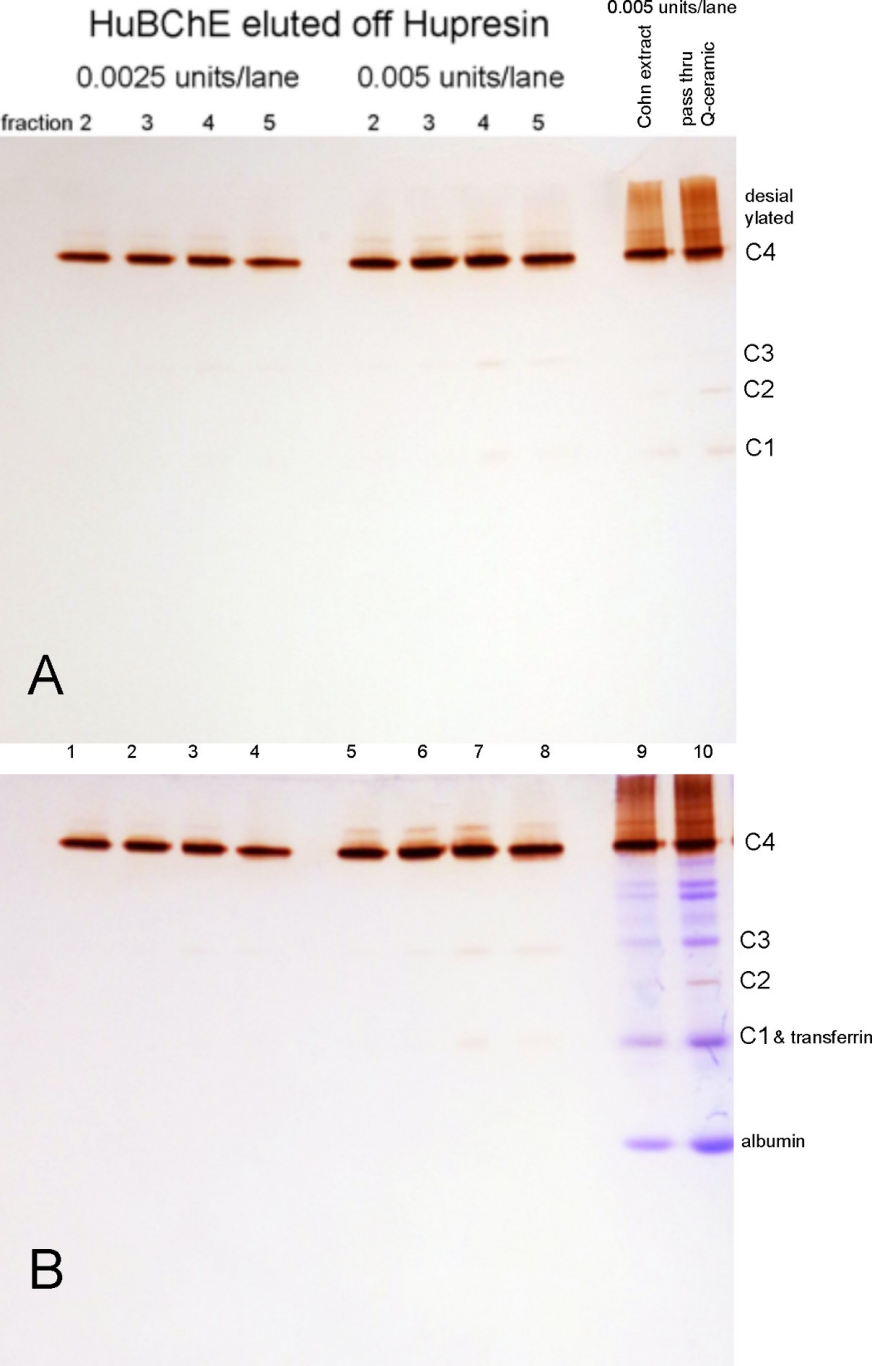
**Nondenaturing polyacrylamide gradient gel stained for BChE activity (A) and counterstained with Coomassie blue (B).** HuBChE eluted from Hupresin (lanes 1–8) consists predominantly of tetramers (C4). Desialylated HuBChE in the Cohn extract and the Q-Ceramic pass-thru migrates near the top of the gel in a broad band (lanes 9 and 10). Cohn paste extract (lane 9) contains partly desialylated HuBChE. The desialylated HuBChE did not bind to Q-Ceramic at pH 4.5, but passed through as visualized by the intense staining for the Q-Ceramic pass through (lane 10). Faint bands of activity are best seen in panel A where C1 is the HuBChE monomer, C2 is an albumin-HuBChE dimer [25], and C3 is a HuBChE dimer. The major band of HuBChE activity is the tetramer C4. The gel counterstained with Coomassie blue (panel B) shows the same pattern of blue bands in lanes 9 and 10. This means the major protein contaminants in the Cohn extract (lane 9) pass through Q-Ceramic without binding (lane 10).

The HuBChE tetramer is sugar coated with a total of 36 N-linked glycans per tetramer [18]. The structures of the glycans have been determined [24]. About 70% of the glycan chains terminate in a negatively charged sialic acid (N-acetylneuraminic acid), thus accounting for the low isoelectric point of 4.4 for the BChE tetramer. Some of the HuBChE in Cohn paste extract is partly desialylated as visualized in Fig 9A lane 9 by the broad band of BChE activity that migrates more slowly than the C4 tetramer. The desialylated molecules do not bind to Q-Ceramic at pH 4.5, but pass through during the sample loading step as seen in lane 10. The pass through fraction also includes a substantial amount of BChE tetramers (C4). The Cohn paste extract and the Q-Ceramic pass-through contain HuBChE monomers (C1), HuBChE-albumin dimers (C2), and HuBChE dimers (C3). The HuBChE eluted off Hupresin (Fig 9A and 9B lanes 1–8) consists predominantly of tetramers (C4).

The gel in Fig 9 panel A was counterstained with Coomassie blue as shown in panel B. Comparison of lanes 9 and 10 in panel B shows that the major protein contaminants in the Cohn extract are also in the pass-through sample (lane 10), indicating they did not bind to the Q-Ceramic sorbent at pH 4.5. The weak C1 band of BChE activity in panel B coincides with a strong band for transferrin protein.

**Fig 10:**
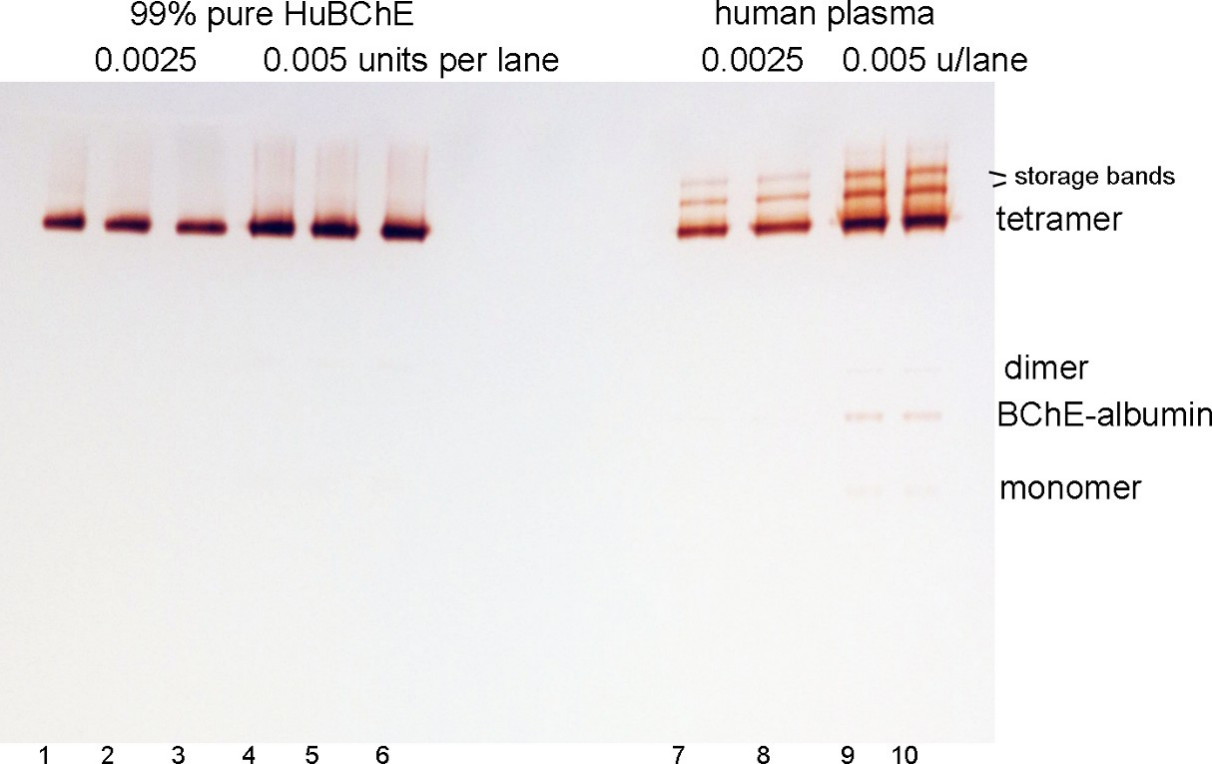
Pure HuBChE from Cohn fraction migrates to the same position as BChE tetramers in human plasma on a nondenaturing gel stained for BChE activity.

The tetramer and sialylation status of purified HuBChE from Cohn fraction were compared to that of BChE in human plasma by visualizing their relative migration in Fig 10. The 99% pure HuBChE from Cohn fraction (lanes 1–6) migrated to the same position as the C4 tetramer in human plasma (lanes 7–10). This indicates the 99% pure HuBChE from Cohn fraction is a tetramer and its sialylation status is the same as that of the BChE tetramer in human plasma. The plasma samples, but not the 99% pure HuBChE, had slow migrating storage bands (lanes 7–10). Storage bands appear in plasma that has been stored [26], but are absent in fresh plasma [27].

### Endotoxin

The endotoxin concentration in the filter-sterilized 25 mg/mL HuBChE solution was 2 EU/mL.

### Amino acid sequence of pure HuBChE and presence of the K-variant

The deglycosylated, carbamidomethylated HuBChE was digested with trypsin and analyzed by LC-MS/MS. The observed peptides covered 99% of the 574 amino acids of the mature, secreted HuBChE sequence in Uniprot accession number P06276. All 9 N-glycosylation sites were confirmed at Asn 17, 57, 106, 241, 256, 341, 455, 481, and 486 [18]. Glycosylation sites were identified by the 1 dalton increase in mass when Asn was converted to Asp by treatment with PNGase F during removal of N-linked glycans.

A common genetic variant, Ala539Thr [28] known as the K-variant, was identified in peptide VLEMTGNIDEA_539_EWEWK. The peptide count for the wild-type sequence was 503 and for the Ala539Thr mutation was 116. This result is consistent with the fact that Cohn paste was prepared from pooled human plasma. It is estimated that 80 kg Cohn paste represents 8000 plasma donors. The frequency of the homozygous K-variant is 1 out of 69 individuals, and the frequency of heterozygous carriers is 1 out of 4. The K-variant of HuBChE, named in honor of Werner Kalow, is a quantitative variant. Human plasma containing the homozygous K-variant of HuBChE has 33% lower activity compared to wild-type [29]. When one BChE allele has the K-variant and the second BChE allele has the wild-type sequence, the average plasma activity is 20% lower, though the broad range of normal activity makes the K-variant indistinguishable on the basis of activity assays [30]. HuBChE mutations that occur at a lower frequency were not identified by LC-MS/MS.

The polyproline-rich peptides that are embedded in the C-terminal tetramerization domain of HuBChE tetramers were identified by mass spectrometry and are published [21].

### Isoelectric point of HuBChE

**Fig 11:**
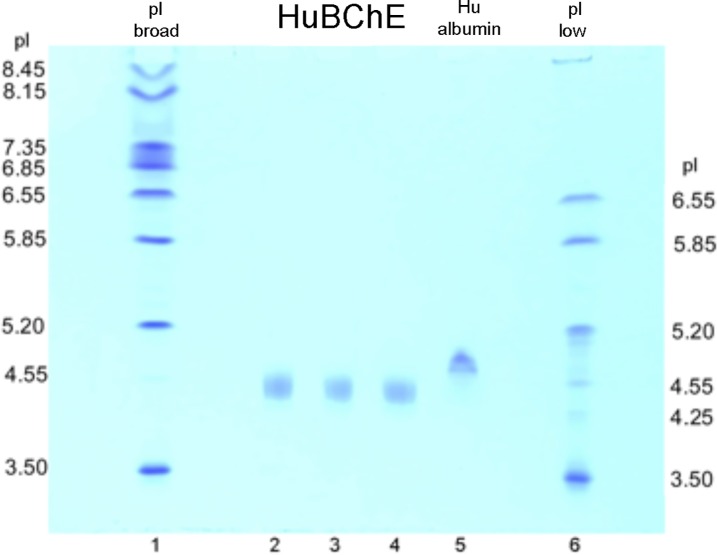
Isoelectric focusing polyacrylamide gel for 3 preparations of HuBChE and for human albumin. Calibration standards are in lanes 1 and 6. Pure HuBChE samples 1 μg each, are in lanes 2, 3, and 4. Human albumin is in lane 5. HuBChE has pI 4.4.

Three preparations of pure HuBChE were loaded at 1 μg per lane in lanes 2, 3, and 4 of the isoelectric focusing polyacrylamide gel in Fig 11. The HuBChE preparations had a pI of 4.4. The human albumin pI of 4.7 is consistent with the literature for undefatted albumin [31].

### Problem of protein aggregation

HuBChE solutions of 25 mg/mL remained clear when the Hupresin eluent was concentrated in the Pellicon from 19 L to 0.1 L. However, the buffer exchange step in the Pellicon where 50 mL of HuBChE were diluted with 100 mL PBS and reconcentrated 15 times resulted in turbidity. Solids were removed by centrifugation in a table top centrifuge at 4000 rpm (2683xg), followed by filtration through a 0.45 μm centrifugal filter, and finally pumped through a 0.22 μm sterile filter. The sterile, clear HuBChE solution in PBS at a concentration of 25 mg/mL became slightly turbid during storage at 4°C after 3 months, but was clear again after 6 months.

In contrast when HuBChE purified from outdated human plasma was concentrated and diafiltered in an Amicon stirred cell, the HuBChE remained clear for years at 4°C. This statement applies exclusively to HuBChE preparations that were never turbid. A HuBChE preparation that became turbid during UF/DF, but was later clarified and concentrated in an Amicon stirred cell, became slightly turbid during storage at 4°C. We interpret this to mean that filtration through a sterile 0.22 μm filter had not removed nucleation sites for growing aggregates.

We suspect that aggregation was promoted by exceedingly high protein concentrations accumulating on the Pellicon membrane surface, resulting in sheer deformation of the HuBChE protein. Stirring in the Amicon stirred cell kept the HuBChE concentration uniform, preventing the high protein concentrations that caused protein to aggregate in the Pellicon.

### Lyophilization buffer

The buffer for lyophilization of HuBChE was 52 mM L-Histidine pH 6.0, 240 mM sucrose, 0.3 mM Tween-20. This buffer was chosen because L-Histidine maintains a constant pH during freezing and thawing, unlike phosphate buffer whose components freeze and thaw at different temperatures resulting in a pH shift that could denature the enzyme. The low pH of 6.0 was selected to minimize deamination of Asn and Gln. The buffer is free of sodium chloride because its presence increases drying time. The pH of L-Histidine HCl was adjusted with L-Histidine in place of sodium hydroxide to avoid producing sodium chloride. Sucrose was added because sucrose stabilizes protein through hydrogen bond contacts. The surfactant Tween-20 was added to protect from aggregation. Two examples of FDA approved proteins for use in humans are lyophilized in a similar buffer–Xolair (Omalizumab) from Genentech/Novartis, and Raptiva (Efalizumab) from Genentech.

### Stability of pure HuBChE solutions stored at 4°C

A 15 mg/mL sterile solution of HuBChE in PBS pH 7.4 stored in a glass lyophilization vial capped with a rubber septum and a metal seal had a specific activity of 500 u/mg, and activity of 7500 u/mL in the year 2012 and the same specific activity of 500 u/mg and 7500 u/mL after being stored for 6 years at 4°C. Filter sterilized HuBChE solutions in PBS with concentrations of 2.3 mg/mL, 6.8 mg/mL, and 16.1 mg/mL stored in sterile glass or plastic bottles in volumes of 50 to 855 mL lost no activity when stored at 4°C for 1 to 3 years.

In contrast, aliquots diluted to 0.2 mg/mL in 1 mL of 20 mM TrisCl pH 8.5, 0.1% sodium azide or 10 mM ammonium bicarbonate pH 8, 0.1% azide in plastic 1.5 mL microfuge tubes lost 20 to 30% activity in 3 years. The loss was explained by HuBChE binding to the walls of the microfuge tubes. Binding was visualized by the bright yellow color that developed when Ellman reagent and butyrylthiocholine were added to the empty, rinsed tubes. This indicated that HuBChE was active when bound to the surface of the plastic tubes.

Concentrated HuBChE also bound to the walls of the glass and plastic containers, but the small amount that adhered to the walls was insignificant compared to pipetting reproducibility and could not be detected as a loss of activity.

### Stability of lyophilized HuBChE

**Fig 12:**
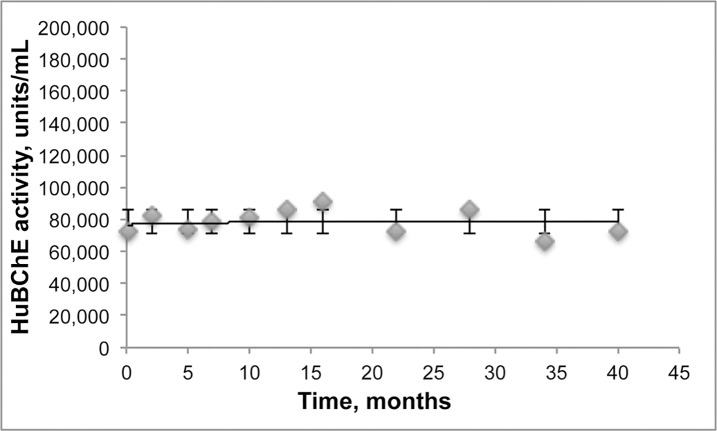
HuBChE lyophilized from 0.1 mL of 144 mg/mL and stored dry at 27°C for up to 40 months was reconstituted by addition of water. The lyophilized HuBChE was stable as a dry powder over the 40 month storage period.

Aliquots of HuBChE in lots of 14.4 mg at a concentration of 144 mg/mL were lyophilized in pH 6.0 buffer and stored at 27°C. Upon reconstitution of the dry powder with water, the BChE activity was found to be unchanged from the original 72,000 units/mL. Fig 12 shows the lyophilized HuBChE was stable at 27°C for at least 40 months.

## Discussion

### Only very fresh, never frozen Cohn paste has high HuBChE activity

Saxena et al. extracted 14 g of HuBChE from 80 kg of Cohn paste prepared by Medimmune [3], and recovered 6 g of highly purified HuBChE. According to personal communication from A. Saxena, this high yield was possible only when paste was very fresh and had never been frozen. Medimmune Inc. is no longer in the plasma fractionation business. We purchased Cohn paste from Grifols Inc. Grifols releases frozen paste after it has been certified free of human pathogens, a process that takes 45 days. We extracted 8.6 g of HuBChE from 80 kg of frozen Grifols paste, and recovered 1.5 g of highly purified HuBChE. Paste stored at -80°C for three years yielded the same amount of pure HuBChE as paste stored for three months. However, paste stored at -20°C for 1 year lost 90% of its HuBChE activity.

### 60% of the HuBChE passed through Q-Ceramic at pH 4.5 without binding

Loss of HuBChE from the Q-Ceramic column was not due to saturation of the sorbent. This is indicated by the fact that the activity passing through the column increased to about 60% as the first 200 liters of extract were loaded, then remained constant for the next 400 liters. If the quantity of HuBChE were saturating the binding capacity of the Q-Ceramic column, then the HuBChE activity in the unbound, pass through material would have increased continuously as more extract was pumped onto the column

Attempts to recover this unbound activity included 1) re-passage over fresh Q-Ceramic at pH 4.5, 2) passage over Q-Sepharose at pH 4.5, and 3) passage over Hupresin. All attempts were unsuccessful. When the unbound material was applied to a regenerated Q-Ceramic column the yield and purity were too low to be cost effective. About 25% of the pass through HuBChE activity was recovered by a second passage over Q-Ceramic at pH 4.5, but the purity was only 0.2%, compared to a purity of 4% for the 1^st^ Q-eluate. Application of the pass through HuBChE onto Hupresin showed that HuBChE failed to bind until the pH was raised to pH 8. This contrasts with the HuBChE in Q-eluate which bound to Hupresin at pH 5.3.

The isoelectric point of 4.4 means the HuBChE tetramer has a net zero charge at pH 4.4. The negatively charged glycans (pKa of neuraminic acid is 2.6) are balanced by positively charged groups on the surface of the protein. At pH 4.5, only a small portion of the BChE has the net negative charge that is required for binding to Q-Ceramic. It is suggested that improved binding and recovery of BChE could be achieved by performing anion exchange chromatography at pH 7 to 8.

### Fold purification

When the starting material is human plasma, BChE has to be purified 15,000-fold to achieve 99% purity [32]. The HuBChE extracted from Cohn fraction IV-4 required only 1000-fold purification to become 99% pure. This shows the HuBChE in Cohn fraction IV-4 is significantly enriched by the plasma fractionation process.

### HuBChE suitable for animal studies, but not human studies

Our purification protocol does not produce GMP quality HuBChE suitable for use in humans. We did not treat the Cohn extract with Triton X-100 and tri(n-butyl)phosphate to inactive virus [3], did not treat with aerosil to remove lipids, did not use GMP quality Hupresin, and did not perform our studies in a certified GMP facility. Furthermore, we did not attempt to minimize the level of prekallikrein activator, a potential thrombogenic agent. We did test for endotoxin in the 99% purified HuBChE and found the level to be 2 EU/mL, suitable for animal studies. Our purified HuBChE had the same pharmacokinetic properties in mice (personal communication, Dr. Douglas Cerasoli) as preparations of HuBChE produced by Saxena et al. [33]. It was concluded that HuBChE purified under our conditions is suitable for studies in animals.

### Stability of lyophilized HuBChE

Saxena et al [34] measured the shelf life of lyophilized HuBChE stored at 4°C, 25°C, 37°C, or 45°C. The buffer for lyophilization was 25 mM sodium phosphate pH 8.0 [5]. The enzyme activity was stable in lyophilized HuBChE stored at 4°C and 25°C for over 2 years. HuBChE reconstituted from lyophilized enzyme stored at -20°C for >3 years [34] had pharmacokinetic properties in mice indistinguishable from those of freshly prepared, never lyophilized HuBChE. Our results confirm that lyophilized HuBChE stored at 27°C for over 3 years retains full activity.

### Isoelectric point pI 4 to 4.4

HuBChE with its isoelectric point of 4.4 is negatively charged at pH 4.5, allowing some of the HuBChE to bind to the positively charged anion exchange sorbent at pH 4.5, while other proteins pass through without binding [35]. HuBChE purified from Cohn fraction IV-4 has a pI of ~4.4, consistent with pI 4.0 values reported for HuBChE purified from human plasma [35, 36]. Removal of sialic acids with neuraminidase shifts the isoelectric point to pH 6.7–7.0 [37]. Desialylated HuBChE is rapidly cleared from the circulation. Asialo receptors in the liver recognize molecules depleted of sialic acid and remove them from blood. A normal complement of sialic acids ensures a long residence time in the circulation. The half-life of HuBChE in the human circulation is 11–16 days [38–40]. Glycoprotein characterization of HuBChE purified from Cohn fraction IV-4 found a molar sialic acid (Neu5Ac) to HuBChE tetramer ratio of about 50 [6, 24], a result consistent with the report that 32.8% of the biantennary glycans are capped by one sialic acid, and 47% are capped by 2 sialic acids.

### Hupresin outperforms procainamide-Sepharose in some applications

Ion exchange chromatography followed by affinity chromatography on either Hupresin or procainamide-Sepharose, yields 99% pure HuBChE when the starting material is Cohn fraction IV-4. However, for some applications Hupresin outperforms procainamide-Sepharose. An example of the superior performance of Hupresin is the observation that HuBChE obtained by simply passing plasma over Hupresin was sufficiently purified to allow detection of the nerve agent modified active site peptide of HuBChE by mass spectrometry [41].

In contrast passage of human plasma through procainamide gel failed to yield detectable levels of the active site peptide. Neither Hupresin nor procainamide gel is capable of purifying BChE from plasma to homogeneity in a single step. Passage of 100 mL human plasma over 2 mL Hupresin yielded 10–15% pure HuBChE [17]. Single-step purification of recombinant HuBChE expressed in insect cells [11] or Chinese Hamster Ovary cells [42] was achieved by chromatography on Hupresin, but not on procainamide gel. Before Hupresin was invented, it was necessary to purify recombinant HuBChE using 3 chromatography steps: procainamide affinity, anion exchange, and a final procainamide affinity chromatography [43].

**Fig 13:**
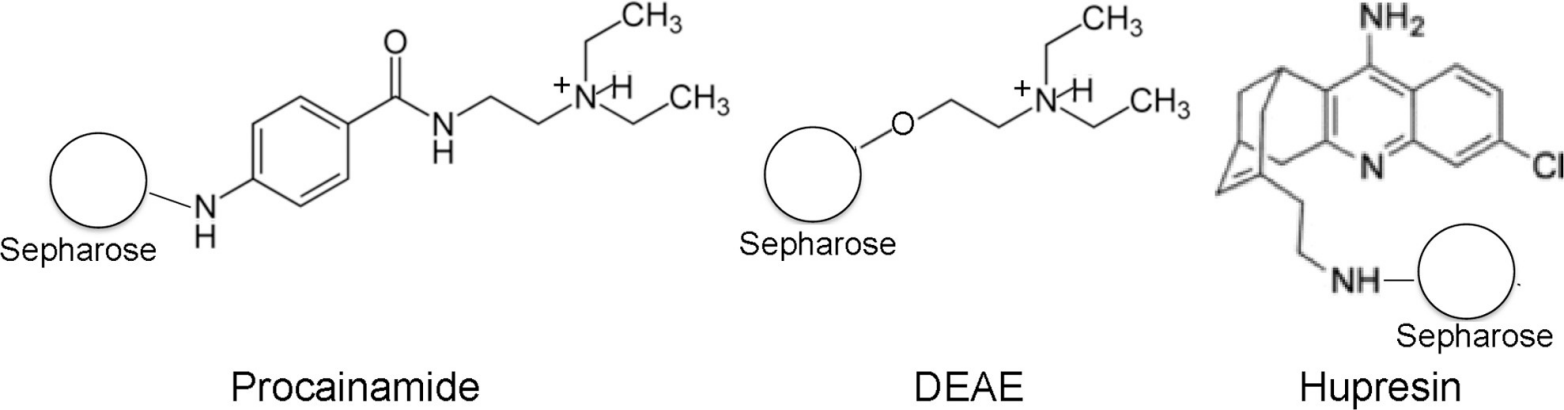
The diethylaminoethyl structure in procainamide is identical to that in the standard DEAE anion exchanger. Hupresin does not have anion exchange properties.

We have found that the binding capacity of procainamide-Sepharose depends on the purity of the sample [10], the purer the sample the more HuBChE could be bound. We believe that this is due to an anion exchange property of procainamide-Sepharose. Fig 13 compares the structures of procainamide, the diethylaminoethyl anion exchanger (DEAE), and Hupresin. Procainamide has the same diethylaminoethyl structure as the DEAE anion exchanger. Thus, procainamide can act as an anion exchanger, and is not simply an affinity gel for HuBChE. The anion exchange property of procainamide explains why this affinity gel binds proteins other than HuBChE, and why its binding capacity depends on the purity of the HuBChE sample. Proteins that are bound through ionic interactions compete with BChE. Purer preparations offer less competition thereby allowing more BChE to bind. The structure of Hupresin indicates that Hupresin does not have the properties of an anion exchanger, consequently contaminant proteins are not effective competitors.

## Conclusion

Frozen Cohn fraction IV-4 has an enriched content of plasma-derived HuBChE, making it suitable as a starting material for purifying HuBChE. Chromatography on Q-Ceramic ion exchange sorbent at pH 4.5 followed by passage through Hupresin yields 99% pure HuBChE.
